# Rate of Vaginal Cuff Dehiscence When Using Vicryl (Poliglactyn 910) Compared to PDS (Polydioxanone) for Vaginal Cuff Closure in Laparoscopic Hysterectomy

**DOI:** 10.3390/medicina60010090

**Published:** 2024-01-03

**Authors:** Francesco Giuseppe Cannone, Livia Cormaci, Carla Ettore, Ferdinando Antonio Gulino, Giosuè Giordano Incognito, Domenico Benvenuto, Giuseppe Ettore

**Affiliations:** 1 Department of Obstetrics and Gynaecology, Azienda di Rilievo Nazionale e di Alta Specializzazione (ARNAS) Garibaldi Nesima, 95122 Catania, Italy; francesco.cannone@hotmail.it (F.G.C.); livia.cormaci23@gmail.com (L.C.); carla.ettore@hotmail.it (C.E.); giordanoincognito@gmail.com (G.G.I.); giuseppe.ettore@gmail.com (G.E.); 2 Unit of Medical Statistics and Molecular Epidemiology, University Campus Bio-Medico of Rome, 00128 Rome, Italy; domenicobenvenuto95@gmail.com

**Keywords:** vaginal cuff dehiscence, Poliglactyn 910, Vicryl, Polydioxanone, laparoscopic hysterectomy, vault closure

## Abstract

*Objective:* To compare the vaginal cuff dehiscence (VCD) rates using Vicryl (Poliglactyn 910) and Polydioxanone (PDS) in patients who underwent laparoscopic hysterectomy. *Materials and methods:* A retrospective, monocentric study was conducted, including all patients undergoing laparoscopic hysterectomy at the Department of Obstetrics and Gynaecology, Azienda di Rilievo Nazionale e di Alta Specializzazione (ARNAS) Garibaldi Nesima, Catania, between January 2014 and December 2021. Patients underwent hysterectomy for benign gynecologic pathologies (endometriosis, leiomyomas, or benign pelvic pathologies) or malignant gynecologic pathologies (endometrium cancer, complex endometrial hyperplasia, ovarian cancer, cervix cancer, or uterine carcinosarcoma). The Z-score calculation was performed to find eventual statistically significant differences between the two populations regarding VCD rates. *Results:* Laparoscopic vaginal cuff closure was performed, with Vicryl sutures in 202 patients and PDS sutures in 184 women. Demographic and baseline characteristics were not significantly different in the two groups. VCD occurred in three patients in the Vicryl group and did not occur in the PDS group. The three cases of VCD were precipitated by intercourses that occurred within 90 days of surgery. However, there was not a significant statistical difference between the two groups regarding VCD (*p* = 0.09). *Conclusions:* Vicryl and PDS sutures seem to be similar for vaginal cuff closure in laparoscopic hysterectomy. The VCD rate was low, and the observed differences between the Vicryl and PDS groups did not reach statistical significance. Further research through prospective studies is essential.

## 1. Introduction

Vaginal cuff dehiscence (VCD) is defined as the partial or complete reopening of a vaginal cuff that was previously closed during a hysterectomy. This condition is considered an uncommon yet serious postoperative complication, with its incidence estimated to be between 0.10% and 1.14% [1]. The rarity of VCD contrasts with its significant clinical implications, necessitating a deeper understanding of its pathophysiology, risk factors, and management strategies. Patients with VCD may present with a range of symptoms that can vary in severity. The most common presentation includes sudden and severe abdominal pain, which can be distressing and debilitating. Dyspareunia, or painful sexual intercourse, is another symptom that significantly affects the quality of life. Additionally, patients may experience vaginal discharge or bleeding, which can be alarming and lead to further complications if not promptly addressed. Systemic symptoms such as fever and nausea are also observed and can be indicative of more severe underlying issues [2,3,4]. In the most extreme cases, VCD can lead to partial or total evisceration of intrabdominal organs, a condition that requires immediate medical attention due to its life-threatening nature. The management of VCD can vary, based on the severity of the condition. Mild cases may be amenable to conservative management, which includes close monitoring and non-surgical interventions. However, due to the potential risks associated with VCD, such as evisceration, bowel strangulation and necrosis, acute mesenteric ischemia, and sepsis, many cases must be treated as surgical emergencies. Vaginal evisceration, characterized by the complete protrusion of abdominal organs through a disrupted vaginal cuff, is a particularly dire emergency that necessitates immediate comprehensive management. This management typically involves patient assessment, stabilization, and surgical repair of the defect [2,3,4]. The timing of VCD occurrence post-hysterectomy varies, based on the surgical technique employed. A study reported a median interval of 11 weeks between the initial hysterectomy and the onset of dehiscence, with cases occurring anywhere from 1 to 13 months post-surgery [5]. This variability underscores the need for prolonged vigilance in the postoperative period.

In recent times, laparoscopic hysterectomy has emerged as the preferred method for this procedure, given its numerous advantages. These benefits include reduced blood loss, shorter recovery time, less postoperative pain, and enhanced patient satisfaction compared to traditional methods. However, it is important to note that studies have indicated a higher incidence of VCD in minimally invasive hysterectomy techniques, such as laparoscopic and robot-assisted procedures. These techniques have been associated with a VCD incidence rate of 1.14%, compared to 0.14% for transvaginal and 0.10% for laparotomic hysterectomies [4,5,6]. A systematic review further corroborated these findings, reporting a 5 to 10 times higher incidence of VCD in minimally invasive surgeries, compared to vaginal or laparotomic methods [7]. 

Several surgical risk factors have been identified concerning VCD. These include the type of surgery performed, the technique used for colpotomy, and the procedure for cuff closure. These factors are crucial in understanding and mitigating the risk of VCD [8]. Additionally, the choice of suture material in vaginal cuff closure has been a topic of debate in the scientific community. The impact of different suture materials on the incidence of VCD remains an area requiring further investigation. Two commonly used suture materials in vaginal cuff closure are Vicryl (Poliglactin 910) and Polydioxanone (PDS). Vicryl is a braided, synthetic, absorbable suture material, effective for approximately two to three weeks and fully absorbed within 8–10 weeks. On the other hand, Polydioxanone is a monofilament suture that, while initially having less tensile strength than Vicryl, retains its strength for a longer period and is fully absorbed in about six months. These distinct properties have led to their differential use in surgical applications, with Vicryl typically preferred for short-term tissue approximation and PDS for situations requiring prolonged support.

The current study aimed to explore and compare the risk of cuff breakdown when using Vicryl versus PDS in vaginal suturing following a laparoscopic hysterectomy. This investigation offered a valuable opportunity to determine if there is a more effective type of suture material in reducing complications after a laparoscopic hysterectomy. By analyzing outcomes associated with these two suture materials, the study endeavored to contribute to the optimization of surgical techniques and the improvement of patient outcomes in hysterectomy procedures.

## 2. Material and Methods

### 2.1. Study Design 

A retrospective, monocentric cohort study was conducted at the Unit of Obstetrics and Gynaecology of the Maternal-Child Department of Azienda di Rilievo Nazionale e di Alta Specializzazione (ARNAS) Garibaldi Nesima in Catania (Italy), including all patients who underwent a laparoscopic hysterectomy during the period between January 2014 and December 2021. The study conformed to the standards contained in the Strengthening the Reporting of Observational Studies in Epidemiology (STROBE) Statement, available through the Enhancing the Quality and Transparency of Health Research (EQUATOR) Network. In addition, all stages of the study respected the guidelines provided by the Helsinki Declaration. Each woman signed an informed consent form for the surgical procedure and data collection. The study was approved by the Ethics Committee of ARNAS Garibaldi Nesima, Catania (Protocol Code 263/C.E approved on 11 May 2022) (Report No. 69/2022/CECT2).

### 2.2. Study Population

All patients who underwent hysterectomy were evaluated. The inclusion criteria included the following: laparoscopic hysterectomy due to benign gynecologic pathologies (endometriosis, benign pelvic pathologies, or leiomyomas) or premalignant and malignant gynecologic pathologies (complex endometrial hyperplasia, endometrial cancer, uterine carcinosarcoma, cervix cancer, or ovarian cancer); age ≥ 18 years; and elective surgery. Exclusion criteria were the following: laparotomic or vaginal hysterectomy; previous vaginal surgery; conversion to laparotomy before the end of the procedure; emergency surgery; and non-gynecological pathologies.

### 2.3. Methods

Retrospective data from the medical charts were collected through a checklist, reviewing demographic characteristics (age, body mass index, parity, premenopausal and postmenopausal status, and prior surgeries), indications for surgery, operative notes (type of hysterectomy and operative time), type of suture, and VCD rates. All laparoscopic hysterectomies were performed by the same surgeon and the respective team, with experience in gynecological laparoscopy > 10 years. The surgeon operated using different techniques, according to the type and gravity of pathology. For endometriosis, benign pelvic pathologies, uterine leiomyomas, and complex endometrial hyperplasia, a total hysterectomy and bilateral adnexectomy were performed. Women with endometrial cancer had radical hysterectomy type A or B1 and bilateral adnexectomy. Uterine carcinosarcoma was treated with radical hysterectomy type A. Cervical cancer was treated by radical hysterectomy type B1, B2 or C1. Laparoscopic hysterectomy for ovarian cancer was performed only for the restaging of cancer in patients without a clear peritoneal impairment. Transvaginal cuff closure was performed using either absorbable Vicryl or PDS sutures. From January 2014 to December 2018, vaginal cuff closure was performed by Vicryl suture. After that period, it was performed by PDS suture. Laparoscopic knots were tied intracorporeally. The same route of hysterectomy and the same technique for cuff closure were performed, applied by the laparoscopic expertise of the surgeon assessed according to the Gynecologic Endoscopic Surgical Education and Assessment (GESEA) program: i.e., laparoscopic hysterectomy with an ultrasonic scalpel device and laparoscopic cuff closure with suspension to the endopelvic fascia and the uterosacral ligaments. Colpotomy was performed by THUNDERBEAT Open Extended Jaw (Olympus^®^, Tokyo, Japan). Vaginal closure was performed with a single-layer running suture. All patients received cefazolin 2 g iv 1 h before surgery, indwelling urinary catheters, and low-molecular-weight heparin (enoxaparin sodium, 4000 UI/die) as thromboembolism prophylaxis postoperatively for 7 days. All patients underwent standardized follow-up at 6 weeks post-operation, including gynecological examination and transvaginal sonography, and were interviewed by telephone about the occurrence of VCD. Patients were instructed to abstain from intercourse for 8–12 weeks postoperatively. All postoperative events, including VCD and evisceration, were recorded. Univariate and multivariable analyses were performed to identify independent predictors of VCD after laparoscopic hysterectomy. For each item of data collected within the study, the mean and the standard deviation were calculated for continuous data, while prevalence percentage was calculated for dummy variables. The sample size calculation was performed in order to verify the margin of error, imposing an alpha of 0.05, a power of 80%, and a prevalence of the condition analyzed of 1%. The Z-score calculation and the chi-squared calculation were carried out to determine eventual statistically significant differences between the two populations. The statistical analysis was performed using MedCalc^®^ Statistical Software version 20.218 (MedCalc Software Ltd., Ostend, Belgium; https://www.medcalc.org; accessed on 1 September 2023).

## 3. Results

A total of 510 women underwent hysterectomy and were evaluated for eligibility criteria. Sixty-two patients were excluded, as they had laparotomic surgery; 40 women with a prolapse of pelvic organs that was treated by a vaginal approach were not considered; 12 patients had previous vaginal surgery; and 10 women were lost during follow-up because they did not come back for gynecological follow-up after surgery or did not answer the standard interview 6 weeks after surgery. Finally, 386 women fulfilled the inclusion criteria and were included in the study. The sample-size calculation showed that the margin of error estimated for this study was less than 0.01.

Transvaginal cuff closure was performed using absorbable Vicryl sutures in 202 women and PDS sutures in 184 women.

Demographic and operative characteristics were comparable in the two populations, as shown in Table 1.

The surgical procedures performed are detailed in Table 2. The most commonly executed surgeries were total hysterectomy + bilateral adnexectomy and radical hysterectomy type A + bilateral adnexectomy, comprising 292 cases (75.6%) of the interventions.

Postoperative VCD were classified according to the pathology treated and the type of suture used, and the results are shown in Table 3.

The largest group was represented by women with endometrial cancer in both groups. VCD occurred in three patients in the Vicryl group (1.5%) and no patients in the PDS group. Specifically, one case of VCD was in a woman undergoing hysterectomy for endometrial cancer and two cases of VCD were in women undergoing hysterectomy for cervical cancer; each case was precipitated by intercourse that occurred at 61, 73, and 80 days post-surgery, respectively. However, there was no significant statistical difference between the two groups in terms of VCD incidence (*p* = 0.09).

## 4. Discussion

This study compared the incidence of VCD among patients undergoing laparoscopic hysterectomy using two different suture materials, Vicryl and PDS, with the aim of identifying the most effective method for reducing postoperative complications.

The authors emphasize that this is a crucial topic, considering that hysterectomy is the second most common surgical procedure after cesarean section occurring in 20–30% of women [9,10]. VCD, while uncommon, is a serious postoperative complication that can occur after a hysterectomy or similar pelvic surgeries. This condition is characterized by either a partial or full separation at the vaginal vault, sometimes leading to evisceration. The earliest recorded case of VCD was documented by Hobbs in 1952 [11]. Further research in 1994 by Somkuti et al. [12] identified multiple factors that could contribute to the weakening of the vaginal apex post-surgery, such as inadequate surgical methods, infection at the surgical site or cuff, hematoma at the wound, premature resumption of sexual activities before complete healing, older age, prior radiation therapy, ongoing steroid use, physical trauma including rape, previous vaginoplasty procedures, and performing Valsalva’s maneuver post-vaginal hysterectomy or during bowel movements. These factors are still largely accepted as relevant today. 

Understanding these risk factors is crucial, especially considering the increased prevalence of laparoscopic hysterectomies, which may elevate the incidence of VCD [4,5,6,7,8,9,10,11,12,13]. The type of suturing material used is also thought to play a role in the likelihood of developing VCD. This synthetic, often-braided suture is produced by Ethicon Inc.^®^, Somerville, NJ, USA a part of the Johnson & Johnson^®^ family, New Brunswick, NJ, USA. It is designed for smooth passage through tissues, reducing drag, and is noted for its easy handling, consistent tie-down smoothness, and excellent knot stability. 

The coated Vicryl suture is recommended for general soft tissue approximation and/or ligation, including ophthalmic procedures, but it is not suitable for cardiovascular and neurological tissue applications. In tissue, it maintains its tensile strength for about two to three weeks and is completely absorbed through acid hydrolysis in 8 to 10 weeks. 

PDS antibacterial surgical suture is a synthetic, absorbable, and sterile monofilament made of polyester. The empirical molecular formula of the polymer is (C_4_H_6_O_3_)_n_. It is a non-antigenic, pyrogenic polymer and causes only a slight tissue reaction during absorption. PDS is composed of multiple ether-ester units, derived through the ring-opening polymerization of p-dioxanone, a process that utilizes heat and an organometallic catalyst, such as zirconium acetylacetone or zinc L-lactate. Notably, its glass transition temperature ranges from −10 to 0 °C, and it exhibits approximately 55% crystallinity. For suture production, PDS is typically extruded into fibers, although it is crucial to process the polymer at the lowest feasible temperature to prevent spontaneous depolymerization back into its monomer form. The flexibility of this suture material is attributed to the ether oxygen group within its polymer chain. PDS sutures undergo degradation via hydrolysis, and their breakdown products are predominantly excreted in urine, with the remainder processed through the digestive system or expelled as CO_2_. This biomaterial is fully absorbed within six months, causing only a minimal foreign body reaction in the surrounding tissue. Furthermore, PDS materials can be sterilized effectively using ethylene oxide. 

Introduced in 1982, PDS represented a pioneering development as the first monofilament synthetic absorbable suture. The monofilament design of PDS offers several advantages, including easier tissue passage, reduced tissue reactivity, and a lower risk of wound infection. This structure, however, leads to decreased handling and knot strength, attributed to its lower coefficient of friction. In terms of tensile strength, PDS initially shows less strength compared to Polyglactin 910 or Polyglycolic acid. However, its slower resorption rate means that PDS maintains its tensile strength for a longer duration. Specifically, it retains 70% of its original tensile strength at 2 weeks, 50% at 4 weeks, and 25% at 6 weeks. Absorption of the suture is minimal until the 90th postoperative day, with complete resorption occurring approximately after 6 months. This extended period of support makes PDS particularly useful for wounds under greater tension or those requiring prolonged dermal support. In fact, extended dermal support for a minimum of 6 months has been linked to reduced scar spreading. 

PDS has undergone improvements since its initial release, with the introduction of PDS II. This newer version offers enhanced handling characteristics, further augmenting its utility in surgical applications. Several research studies indicated that barbed sutures may be associated with lower rates of vaginal cuff dehiscence (VCD) compared to braided sutures [14] or to both braided and monofilament sutures [15]. Additionally, the use of barbed sutures has been linked to benefits such as reduced operation time, lesser post-operative blood loss, shorter hospital stays, and decreased vaginal cuff granulation six months after the operation [16]. Unidirectional barbed sutures for vaginal cuff closure have been reported as safe, without major complications noted. However, Weizman et al. [17] found that while a continuous suture method was protective against VCD, there was no significant difference between braided and barbed sutures, nor between suturing by hand or laparoscopically. In contrast, Blikkendaal et al. [18] observed no statistical difference in VCD rates when comparing various methods (vaginal or laparoscopic interrupted sutures or laparoscopic running sutures) or types of sutures (braided and barbed). 

In their research, MacKoul et al. [19] compared absorbable and non-absorbable sutures without finding a significant difference in effectiveness, although non-absorbable sutures required surgical removal after 90 days. Uccella et al. [20] conducted a meta-analysis revealing that barbed sutures presented a lower risk of VCD, which was particularly significant when compared to total laparoscopic hysterectomies. This improved outcome might be attributed to the superior tensile strength of barbed sutures compared to polyglactin sutures. They also noted that barbed sutures took longer to lead to dehiscence, compared to polyglactin sutures (73 days vs. 29 days). However, animal studies have shown mixed results. One study in ewes found no difference in adhesion formation between barbed and polyglactin sutures [21], while a similar study in rats observed increased adhesion formation and a greater presence of inflammatory cells with barbed sutures [22]. 

The risk of cuff dehiscence is increased in laparoscopic or robotic hysterectomies, where tissues heal slowly due to the use of thermal energy for hemostasis [5]. Suture type is also implicated in VCD, considering that the surgical knot and the knot’s area are most vulnerable to knot slippage [23]. The absorption process of Vicryl sutures involves both a reduction in tensile strength and mass loss. Studies conducted on rat implants revealed that Vicryl sutures maintain about 75% of their original tensile strength two weeks after implantation. By the third week, this retention drops to around 50% for suture sizes 6-0 and larger, and about 40% for sizes 7-0 and smaller. By the fourth week, the strength retention is approximately 25% for sizes 6-0 and larger. All original tensile strength is typically lost by the fifth week post-implantation. The complete absorption of coated Vicryl sutures usually occurs between 56 and 70 days. 

Two key factors define the in vivo performance of absorbable sutures: tensile strength retention and the rate of absorption (or mass loss). PDS II suture, in particular, has been engineered to minimize the variability in these characteristics, thereby providing consistent wound support over an extended period of healing. Synthetic polymers, such as Vicryl and PDS suture, degrade by hydrolysis. The presence of aliphatic ester bonds in these suture materials renders them hydrolytically unstable; they degrade by hydrolysis from body fluids. Water molecules penetrate the polymer threads and break down the polymer chains of the suture. Synthetic sutures have a predictable loss of tensile strength. Typically, Vicryl suture is fully absorbed by 70 days and PDS II suture is essentially absorbed between 182 and 238 days post-implantation, although this can vary depending on many factors, including the level of hydration. Behbehani et al. [24] evaluated VCD after laparoscopic hysterectomy using absorbable PDS and V-loc sutures or absorbable Vicryl sutures. The authors concluded that a delayed absorbable suture is preferable for vaginal cuff closure at laparoscopic hysterectomy, compared to an absorbable suture. 

Most of the cases of VCD cases occur in the first 90 days after surgery [25,26]. The choice of suture material is related to the complication rate, although there is currently no consensus about the best suture material to use. Therefore, based on the previous studies and the fact that the PDS group did not prove to be inferior to the Vicryl group in terms of VCD rate, the authors believe that using the latter suture, which is absorbed later than the former, could represent a valid option.

In this study, all three cases of VCD were observed in the Vicryl group, with no patients in the PDS group reporting this complication. Given previous research and the fact that the PDS group did not prove to be inferior to the Vicryl one, the authors believe that the former suture type offers a viable alternative. It is important to note that the only instances of VCD occurred in the Vicryl group and were triggered by intercourse within 90 days post-surgery. This would support the non-inferiority of the use of PDS, which has delayed absorption and maintains its tensile strength for a more extended period.

The strengths of the present study include a substantial number of cases, uniformity in the surgical procedure performed by the same surgeon, the same method of colpotomy used in all patients, and consistency in the mode of vaginal cuff closure (performed with a single-layer running suture). The study’s use of advanced surgical techniques and state-of-the-art equipment provides an added layer of precision and reliability to the surgical outcomes. The use of consistent surgical protocols across all cases minimizes intraoperative variability, thus enhancing the study’s internal validity. This approach allowed for a direct comparison between the two different types of suture materials. Moreover, the study’s design minimized variations due to different surgical approaches or operative variables, providing more reliable and replicable results. 

However, the retrospective nature of the study was a limitation, influenced by the accuracy of medical data and transcription errors. Additionally, the variety of physicians following up with the patients post-hysterectomy can introduce variability in outcomes and follow-up practices. This factor makes it challenging to estimate the true incidence of suture-related complications after laparoscopic hysterectomy. Other limitations include the absence of randomization and control groups, which could provide stronger comparisons and further reduce potential biases. The duration of follow-up may also be insufficient to identify all long-term complications. Additionally, the sample’s representativeness might not adequately reflect the general population, limiting the generalizability of the results.

## 5. Conclusions

In conclusion, this study investigated the use of Vicryl and PDS sutures for vaginal cuff closure in laparoscopic hysterectomy, revealing similar outcomes with both materials. Notably, all cases of VCD occurred exclusively with Vicryl sutures. This absence of VCD in the PDS group may be attributed to its slower absorption rate and prolonged tensile strength retention. However, it is important to emphasize that the overall rate of VCD was relatively low, and the differences observed between the Vicryl and PDS groups did not reach statistical significance. These findings suggest that while both suture types are effective for vaginal cuff closure, PDS may offer some advantages in reducing VCD risk, likely due to its longer duration and physical properties retention. This is particularly relevant in a surgical context, where preventing postoperative complications is crucial for patient well-being. Given the importance of suture material choice in surgical practice, further research is needed to confirm these preliminary findings. Large-scale prospective studies could provide more insights into the relationship between suture type and complications like VCD in laparoscopic hysterectomy. Such research could not only help define best surgical practices but also improve outcomes for patients undergoing this procedure.

## Figures and Tables

**Table 1:** Patients and operative characteristics of the two groups.

Clinical Data	Vicryl	PDS	*p*-Value
Patients (*n*, %)	202	184	
Age (years) (mean ± SD)	65.3 ± 8.5	64.9 ± 7	*p* = 0.96
BMI (kg/m^2^) (mean ± SD)	30.7 ± 5.5	30.5 ± 5.4	*p* = 0.98
Parity	2.0 (1.00–3.00)	2.0 (1.00–3.00)	*p* = 0.99
Premenopausal status (*n*, %)	72 (35.6%)	78 (42.4%)	*p* = 0.42
Postmenopausal status (*n*, %)	130 (65.4%)	106 (57.6%)	*p* = 0.47
Prior laparotomy (*n*, %)	117 (57.9%)	79 (42.9%)	*p* = 0.13
Prior laparoscopy (*n*, %)	85 (42.1%)	81 (44.0%)	*p* = 0.92
Any prior surgical history (*n*, %)	87 (43.1%)	78 (42.4%)	*p* = 0.91
Operative time (min) (mean ± SD)	110 ± 10.5	106.5 ± 12	*p* = 0.98
Benign pathology (*n*, %)	55 (27.2%)	47 (25.5%)	*p* = 0.47
Malignant pathology (*n*, %)	147 (72.8%)	137 (74.4%)	*p* = 0.86

Abbreviations: BMI, body mass index; *n*, number; %, percentage. Continuous variables are expressed as mean ± standard deviations (SD) and categorical variables were summarized as percentages. The parity is expressed as the median (Q1, Q3). *p*-value was calculated using the Z-score calculation and chi-squared calculation.

**Table 2:** Type of hysterectomy.

Type of Hysterectomy	Patients (*n*, %)
Total hysterectomy + bilateral adnexectomy	155 (40.1%)
Radical hysterectomy type A + bilateral adnexectomy	137 (35.5%)
Radical hysterectomy type A + partial colpectomy	5 (1.3%)
Radical hysterectomy type B1 + bilateral adnexectomy	57 (14.8%)
Radical hysterectomy type B2 + bilateral adnexectomy	23 (6.0%)
Radical hysterectomy type C1 + bilateral adnexectomy	9 (2.3%)

Abbreviations: *n*, number; %, percentage.

**Table 3:** Postoperative VCD occurrence of the two groups for each gynecological pathology.

Gynecological Pathology	Polyglactin 910 *n* (%)	VCD *n* (%)	PDS *n* (%)	VCD *n* (%)	*p*-Value
Endometriosis	3 (1.5%)	-	1 (0.5%)	-	*p* = 0.96
Benign pelvic pathologies	21 (10.4%)	-	19 (10.3%)	-	*p* = 0.99
Uterine leiomyomas	31 (15.3%)	-	27 (14.6%)	-	*p* = 0.98
Complex endometrial hyperplasia	28 (13.9%)	-	25 (13.6%)	-	*p* = 0.99
Endometrial cancer	73 (36.1%)	1 (0.5%)	70 (38%)	-	*p* = 0.87
Uterine carcinosarcoma	4 (2%)	-	2 (1.5%)	-	*p* = 0.92
Cervix cancer	28 (13.9%)	2 (1%)	27 (14.5%)	-	*p* = 0.96
Ovarian cancer	14 (6.9%)	-	13 (7%)	-	*p* = 0.99
Total	202	3 (1.5%)	184	0 (0%)	*p* = 0.09

Abbreviations: *n*, number; PDS, polydioxanone; VCD, vaginal cuff dehiscence; %, percentage. *p*-value was calculated using the chi-squared calculation.

## Data Availability

Data are contained within the article.
